# Associations of exposure to secondhand smoke with hypertension risk and blood pressure values in adults

**DOI:** 10.1186/s12199-021-01009-0

**Published:** 2021-09-06

**Authors:** Qi Zhang, Guowei Zeng, Xiaowei Wang, Kai-Hong Wu

**Affiliations:** 1 Department of Cardiothoracic Surgery, Children’s Hospital of Nanjing Medical University, 72 Guangzhou Road, Nanjing, 210008 China; 2 Department of Cardiovascular Surgery, The First Affiliated Hospital of Nanjing Medical University, Nanjing, 210029 China

**Keywords:** Secondhand smoking, Hypertension, NHANES

## Abstract

**Background:**

The effects of environmental chemical exposure on blood pressure (BP) have been confirmed, but the association between exposure to secondhand smoke (SHS) and hypertension risk and BP in the general population remains unknown.

**Methods:**

Cross-sectional associations between SHS exposure and hypertension risk and BP values were evaluated using data for subjects who participated in the National Health and Nutrition Examination Survey (NHANES), 1999–2016. Logistic regression and linear regression were performed after adjusting for age, sex, race, alcohol consumption, poverty-to-income ratio (PIR), body mass index (BMI), estimated glomerular filtration rate, physical activity, diabetes, cardiovascular disease, and NHANES cycle. Restricted cubic spline models were created to display the potential nonlinear association between SHS and BP levels.

**Results:**

Higher risk of hypertension was found at the highest SHS concentrations (OR = 1.13, 95% CI 1.04, 1.24, *P* for trend = 0.007). Additionally, SHS exposure had a strong positive association with systolic blood pressure (SBP) but was negatively associated with diastolic blood pressure (DBP). Furthermore, the nonlinear model result showed a significant association between SHS and SBP (*P* = 0.017); however, the nonlinear model result was not significant for SHS or DBP.

**Conclusions:**

Our results suggest a potential association between high SHS exposure and the risk of hypertension. Further research is needed to elucidate the underlying mechanisms.

**Supplementary Information:**

The online version contains supplementary material available at 10.1186/s12199-021-01009-0.

## Introduction

Hypertension or elevated blood pressure (BP) is defined as systolic blood pressure (SBP) higher than 140 mmHg and/or diastolic blood pressure (DBP) higher than 90 mmHg [1]. Hypertension is a global health issue that can result in severe consequences, such as cardiovascular disease (CVD), kidney failure, blindness, stroke, and other noncommunicable diseases [2–6]. According to a study analyzing hypertension patients from 90 countries until 2010, 1.39 billion adults aged 20 years or older were estimated to be diagnosed with hypertension, accounting for 31.1% of the worldwide population [7]. Furthermore, a greater number of hypertension patients was estimated when undiagnosed asymptomatic hypertension and adolescent hypertension were considered [8]. Additionally, the World Health Organization noted that hypertension can be prevented and that its risk factors should receive public attention to promote healthier lifestyle habits [9]. It is widely considered that risk factors for adult hypertension are age, unhealthy diet, tobacco use, physical inactivity, obesity, and others [10]. Environmental factors have also been reported to be nonnegligible factors influencing BP [11, 12], and secondhand smoke (SHS) has been a concern in recent years [13, 14].

SHS, or passive smoke, is an environmental pollutant composed of particulate matter generated by tobacco [15]. The reported sites of exposure to SHS are homes, restaurants, workplaces, and bars [16, 17]. Furthermore, serum cotinine, an assessment of the levels of exposure to nicotine, can also be detected in nonsmokers. A very large number of people suffer from SHS. In Southwest China, more than 70% of people are exposed to SHS, and most people are reluctant passive smokers [18]. Although tobacco policies have been implemented by various countries, many people are still exposed to SHS, especially in younger, lower income, and lower education groups [19]. Many studies have shown that SHS contributes to numerous diseases, including cancer [20–23], depression [24–26], multiple sclerosis [27], and CVDs [28, 29]. However, there is no definite conclusion about the effect of SHS on hypertension and BP.

Previous epidemiological studies have shown that SHS is associated with hypertension risk [13, 14, 30–37], but most of those studies were based on questionnaires, which may be limited by recall bias due to recall error or reporting bias due to subjectivity of the questionnaire survey information. Moreover, it is difficult to quantify SHS exposure via questionnaires, which is another disadvantage. The present study has a larger sample size than previous studies performed to date, including 26,578 participants. Quantitative cotinine data rather than questionnaires were used to assess the degree of SHS exposure. We conducted a stratified analysis and a *P* interaction analysis to identify sensitive populations with regard to the association of SHS and hypertension. In addition, because SBP and DBP are continuous variables, we further evaluated the nonlinear relationship between serum cotinine levels and SBP and DBP.

## Methods

### Subjects

Publicly available data were used in our study. Subjects were recruited from nine cycles of the National Health and Nutrition Examination Survey (NHANES) (1999–2000, 2001–2002, 2003–2004, 2005–2006, 2007–2008, 2009–2010, 2011–2012, 2013–2014, and 2015–2016). The survey design, available data, and methods are detailed on the NHANES website [https://www.cdc.gov/nchs/nhanes/]; NHANES included a sample representative of the noninstitutionalized U.S. population. Biological samples were collected, physical examinations were conducted, and questionnaires were completed in mobile examination centers (MEC).

Subjects who had taken part in the NHANES and whose serum SHS indicator concentrations and BP values were available were recruited. We screened subjects > 15 years of age in our study who had completed both the NHANES interview and the examination; those who had a smoking history or had missing SHS data were excluded. A total of 25,678 people were included in our study.

### Serum cotinine detection

Cotinine is a major metabolite of nicotine, and the half-life of cotinine is longer than that of nicotine (cotinine, 15–20 h vs nicotine, 0.5–3 h). Therefore, cotinine can be used as a marker of environmental tobacco smoke exposure or “passive smoking.” Blood samples were collected at a medical examination center, and the levels of serum cotinine were used to represent the extent of exposure to nicotine, which was measured by isotope dilution high-performance liquid chromatography/atmospheric pressure chemical ionization tandem mass spectrometry (ID HPL-APCI MS/MS). After comparison with the standard curve, the cotinine concentrations were defined according to the ratio of native and labeled cotinine, which is detailed in the NHANES [38]. The distribution of serum cotinine levels is shown in Figure S1.

### Outcome assessment

Three consecutive BP readings were obtained for each participant with a sphygmomanometer by a designated person after the participant rested for at least 5 min. In the present study, we calculated the average of up to 3 brachial systolic (first Korotkoff phase) and diastolic (fifth Korotkoff phase) BP readings for every participant [39]. A trained physician evaluated the BP values and measured the value with quality control measures in place. The methods of assessing BP were consistent from 1999 to 2016. Hypertension was defined as one of the following three indicators according to previous studies [40, 41]: First, a mean SBP ≥ 140 mm Hg or a mean DBP ≥ 90 mm Hg. Second, self-reported use of antihypertensive medication. Third, answering yes to the question “Have you ever been told by a doctor or other health care professional that you had hypertension.”

### Covariate analysis

Information on physical activity was collected by a self-administered questionnaire. Moderate physical activity was defined as a task that results in slight sweating, a slight increase in breathing, or a slight to moderate increase in heart rate. Strenuous physical activity was defined as a task that results in heavy sweating or a significant increase in breathing or heart rate. Incident diabetes was defined as a self-reported physician diagnosis of diabetes or hemoglobin A1c (HbA1c) level ≥ 6.5%. Incident CVD was defined as any positive self-reported physician diagnosis of congestive heart failure, coronary heart disease, angina pectoris, heart attack, or stroke. All subjects completed the questions related to CVDs in the medical condition questionnaire. The estimated glomerular filtration rate (GFR) is used to reflect kidney function. The calculation formula is as follows: estimated GFR = 175 × standardized Scr ^−1.154^ × age^−0.203^ × 1.212 [if black] × 0.742 [if female], referring to the published literature [42]. Because several factors may influence the outcomes, we selected age (continuous variable), sex (categorical variable, male and female), race (categorical variable, Mexican American, other Hispanic, non-Hispanic white, non-Hispanic black and other race including multiracial), alcohol consumption (categorical variable, no and yes), poverty-to-income ratio (PIR, categorical variable, less than 1 and great than or equal to 1), body mass index (BMI, categorical variable, less than 25, 25–30, and greater than or equal to 30), estimated GFR (continuous variable), physical activity (categorical variable, none, moderate and vigorous), incident diabetes (categorical variable, no and yes), incident CVD (categorical variable, no and yes), and NHANES cycle (categorical variable) as the covariates in our analysis models. We extracted specific information related to these variables from questionnaires, the NHANES examination data, and laboratory detection data.

### Statistical analysis

For continuous variables, a normal distribution test was carried out. If the variable was normally distributed, a one-way analysis of variance was used for three groups, and an independent samples *t* test was used for two groups. The Mann-Whitney *U* test was used for nonnormally distributed variables. We used the chi-square test for categorical variables. We performed logistic regression and general linear regression to explore the association between SHS and hypertension risk and BP levels. We conducted restricted cubic spline (RCS) regression to investigate the nonlinear relationship between SHS and BP levels. A *P* value less than 0.05 was considered statistically significant. The statistical analyses were carried out with IBM SPSS software, version 20.0 (IBM Corp., Armonk, NY, USA) and R v3.5.0.

## Results

Table 1 shows the baseline characteristics by SHS tertiles. The participants’ ages were 46.76 ± 20.32 in the lowest SHS group and 37.12 ± 19.91 in the highest SHS group. There were more males in the highest SHS exposure group, but fewer females. Subjects who were non-Hispanic black and had a BMI greater than 30 tended to be exposed to more SHS. The highest SHS exposure group had more participants with a family income index PIR of less than 1. Furthermore, a higher number of people in the highest SHS exposure group participated in vigorous physical activity and did not have diabetes. However, fewer people in the highest SHS exposure group drank and suffered from CVD. The serum cotinine levels (ng/mL) of subjects were 0.01 ± 0, 0.03 ± 0.10, and 0.67 ± 1.30 in the low, medium, and high SHS groups, respectively. The proportions of hypertension were 23.9%, 28.0%, and 29.5% in the three groups, respectively.

**Table 1:** Characteristics of the study population by secondhand smoke exposure category from the National Health and Nutrition Examination Survey, 1999–2016

	Total (*n* = 26578)	Tertile 1 (*n* = 9038)	Tertile 2 (*n* = 8779)	Tertile 3 (*n* = 8761)	*P* value
Age (year)	42.8 ± 20.6	46.8 ± 20.3	44.4 ± 20.4	37.1 ± 19.9	< 0.001
Sex					< 0.001
Male	10789 (40.6%)	3220 (35.6%)	3628 (41.3%)	3941 (45.0%)	
Female	15789 (59.4%)	5818 (64.4%)	5151 (58.7%)	4820 (55.0%)	
Race					< 0.001
Mexican American	6012 (22.6%)	2280 (25.2%)	2117 (24.1%)	1615 (18.4%)	
Other Hispanic	2392 (9.0%)	1009 (11.2%)	761 (8.7%)	622 (7.1%)	
Non-Hispanic white	9828 (37.0%)	3812 (42.2%)	3184 (36.3%)	2832 (32.3%)	
Non-Hispanic black	5709 (21.5%)	1023 (11.3%)	1671 (19.0%)	3015 (34.4%)	
Other race—including multiracial	2637 (9.9%)	914 (10.1%)	1046 (11.9%)	677 (7.8%)	
BMI category (%)					< 0.001
< 25	9373 (35.3%)	3177 (35.2%)	3108 (35.4%)	3088 (35.3%)	
25–30	8556 (32.1%)	2976 (32.9%)	2907 (33.1%)	2673 (30.5%)	
≥ 30	8649 (32.6%)	2885 (31.9%)	2764 (31.5%)	3000 (34.2%)	
Family PIR (%)					< 0.001
< 1	5003 (18.8%)	1287 (14.2%)	1464 (16.7%)	2252 (25.7%)	
≥ 1	21575 (81.2%)	7751 (85.8%)	7315 (83.3%)	6509 (74.3%)	
Physical activity					< 0.001
None	13672 (51.4%)	5033 (55.7%)	4492 (51.2%)	4147 (47.3%)	
Moderate	6025 (22.7%)	2134 (23.6%)	2009 (22.9%)	1882 (21.5%)	
Vigorous	6635 (25.0%)	1810 (20.0%)	2181 (24.8%)	2644 (30.2%)	
Missing	246 (0.9%)	61 (0.7%)	97 (1.1%)	88 (1.0%)	
Estimated glomerular filtration rate (mL/min/1.73 m^2^)	98.2 ± 31.5	92.2 ± 27.6	98.3 ± 32.4	104.3 ± 33.2	< 0.001
Diabetes history					< 0.001
No	24025 (90.4%)	8054 (89.1%)	7924 (90.3%)	8047 (91.9%)	
Yes	2548 (9.6%)	984 (10.9%)	853 (9.7%)	711 (8.1%)	
Missing	5 (0.02%)	0 (0)	2 (0.02%)	3 (0.03%)	
Alcohol consumption					< 0.001
No	8600 (32.3%)	3291 (36.4%)	3044 (34.7%)	2265 (25.8%)	
Yes	11442 (43.1%)	4103 (45.4%)	3836 (43.7%)	3503 (40.0%)	
Missing	6536 (24.6%)	1644 (18.2%)	1899 (21.6%)	2993 (34.2%)	
CVD history					<0.001
No	19598 (73.7%)	7177 (79.4%)	6742 (76.8%)	5679 (64.8%)	
Yes	1801 (6.8%)	658 (7.3%)	628 (7.1%)	515 (5.9%)	
Missing	5179 (19.5%)	1203 (13.3%)	1409 (16.1%)	2567 (29.3%)	
NHANES cycle					< 0.001
1999–2000	2561 (9.6%)	0 (0)	1346 (15.3%)	1215 (13.9%)	
2001–2002	2830 (10.7%)	836 (9.3%)	832 (9.5%)	1162 (13.3%)	
2003–2004	2669 (10.0%)	741 (8.2%)	788 (9.0%)	1140 (13.0%)	
2005–2006	2709 (10.2%)	769 (8.5%)	914 (10.4%)	1026 (11.7%)	
2007–2008	2983 (11.2%)	917 (10.2%)	1010 (11.5%)	1056 (12.1%)	
2009–2010	3306 (12.4%)	1268 (14.0%)	1122 (12.8%)	916 (10.5%)	
2011–2012	3042 (11.5%)	1232 (13.6%)	1043 (11.9%)	767 (8.8%)	
2013–2014	3265 (12.3%)	1648 (18.2%)	864 (9.8%)	753 (8.5%)	
2015–2016	3213 (12.1%)	1627 (18.0%)	860 (9.8%)	726 (8.2%)	
Serum cotinine level (ng/mL)	0.2 ± 0.8	0.01 ± 0	0.03 ± 0.10	0.7 ± 1.3	< 0.001
Hypertension, cases (%)	7218 (27.2%)	2092 (23.9%)	2459 (28.0%)	2667 (29.5%)	< 0.001
Systolic blood pressure (mmHg)	121.7 ± 18.9	120.6 ± 18.2	122.4 ± 19.6	122.2 ± 18.6	< 0.001
Diastolic blood pressure (mmHg)	68.5 ± 13.0	68.5 ± 12.4	69.1 ± 13.2	68.5 ± 13.3	< 0.001

Tertile 1, serum cotinine levels < 0.017 ng/mL. Tertile 2, serum cotinine levels 0.017–0.055 ng/mL. Tertile 3, serum cotinine levels ≥ 0.055 ng/mL

*BMI*, body mass index; *PIR*, poverty-to-income ratio; *CVD*, cardiovascular disease; *NHANES*, National Health and Nutrition Examination Survey

Table 2 shows that the results of the logistic regression analyses suggested that high SHS exposure was associated with increasing hypertension risk (OR = 1.13, 95% CI 1.04, 1.24, *P* for trend = 0.007). In addition, a separate analysis stratified by covariates revealed that subjects who were female, non-Hispanic black, had a PIR greater than 1, with no CVD and no diabetes had the highest SHS exposure and increased hypertension risk, although the *P* values of the interaction terms were greater than 0.05. The effects of the OR of other factors are listed in Table S1.

**Table 2:** ORs and 95% CI for the associations between secondhand smoke concentrations and hypertension risk in the National Health and Nutrition Examination Survey, 1999–2016

	Tertile 1	Tertile 2	Tertile 3	*P* for trend	*P* for interaction
Total	Reference	1.05 (0.96, 1.13)	1.13 (1.04, 1.24)	0.007	
Sex					0.722
Male	Reference	1.08 (0.95, 1.23)	1.11 (0.96, 1.28)	0.169	
Female	Reference	1.02 (0.92, 1.13)	1.16 (1.03, 1.30)	0.017	
Race					0.356
Mexican American	Reference	0.93 (0.77, 1.12)	0.97 (0.78, 1.20)	0.690	
Other Hispanic	Reference	0.87 (0.66, 1.15)	1.20 (0.88, 1.64)	0.404	
Non-Hispanic white	Reference	1.11 (0.98, 1.26)	1.14 (0.99, 1.31)	0.057	
Non-Hispanic black	Reference	1.23 (1.00, 1.52)	1.30 (1.06, 1.59)	0.017	
Other race—including multiracial	Reference	0.86 (0.66, 1.11)	0.98 (0.72, 1.32)	0.780	
BMI category					0.549
< 25	Reference	1.02 (0.86, 1.22)	1.15 (0.94, 1.41)	0.195	
25–30	Reference	0.93 (0.82, 1.07)	1.16 (1.00, 1.35)	0.067	
≥ 30	Reference	1.16 (1.02, 1.32)	1.09 (0.96, 1.25)	0.200	
Family PIR					0.328
< 1	Reference	1.06 (0.84, 1.32)	1.15 (0.92, 1.43)	0.217	
≥ 1	Reference	1.04 (0.95, 1.14)	1.12 (1.01, 1.23)	0.028	
Alcohol consumption					0.808
No	Reference	1.02 (0.89, 1.16)	1.14 (0.98, 1.31)	0.156	
Yes	Reference	1.06 (0.94, 1.19)	1.10 (0.96, 1.25)	0.093	
CVD history					0.288
No	Reference	1.03 (0.94, 1.13)	1.12 (1.02, 1.24)	0.022	
Yes	Reference	1.13 (0.86, 1.49)	1.11 (0.82, 1.50)	0.485	
Diabetes history					0.607
No	Reference	1.04 (0.95, 1.13)	1.14 (1.03, 1.25)	0.011	
Yes	Reference	1.11 (0.89, 1.39)	1.11 (0.87, 1.42)	0.370	
Physical activity					0.745
None	Reference	1.03 (0.92, 1.15)	1.08 (0.96, 1.22)	0.217	
Moderate	Reference	1.05 (0.89, 1.25)	1.20 (1.00, 1.44)	0.051	
Vigorous	Reference	1.09 (0.89, 1.33)	1.22 (1.00, 1.50)	0.054	

*OR*, odds ratio; *CI*, confidence interval; *BMI*, body mass index; *PIR*, poverty-to-income ratio; *CVD*, cardiovascular disease

The results were obtained after adjusting for age, sex, race, PIR, BMI, physical activity, diabetes, estimated glomerular filtration rate, alcohol consumption, CVD, and NHANES cycle

For the National Health and Nutrition Examination Survey, 1999–2016, the results were obtained after adjusting for all covariates except for the corresponding stratification variable

**Fig. 1:**
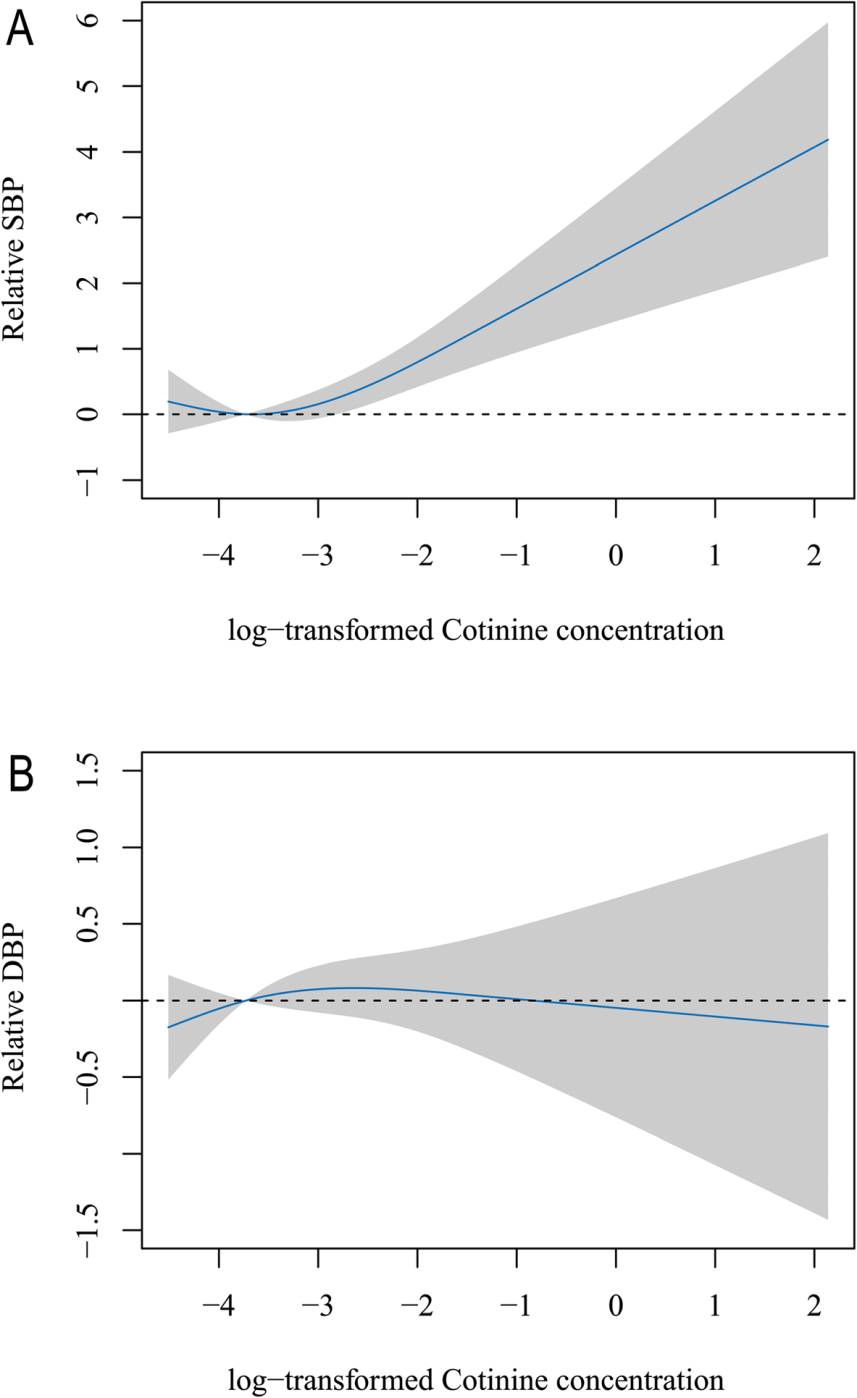
Predicted spline curves for the associations of systolic blood pressure (**A**) and diastolic blood pressure (**B**) with secondhand smoke concentrations according to restricted cubic spline regression models

Table 3 indicates that SBP levels tended to be higher (beta = 0.43, 95% CI 0.18, 0.67) and DBP levels tended to be lower (beta = − 0.31, 95% CI − 0.51, − 0.12) in the highest SHS exposure group. In terms of the association between cotinine and SBP, positive correlations were found for subjects who were female, non-Hispanic black, with a BMI between 25–30, a PIR greater than 1, no exercise or moderate exercise, no diabetes, and no CVD. There was a positive correlation between cotinine and SBP regardless of whether the subject drank or not. For the association of cotinine and DBP, negative correlations were observed for males, Mexican Americans, those with a BMI less than 25 or greater than 30, a PIR greater than 1, no diabetes, and no exercise or vigorous exercise. Figure 1 reveals the continuous associations of SHS exposure with SBP and DBP based on the RCS regression models. A nonlinear association was found between SHS exposure and SBP (*P* value = 0.017), while no association was observed for SHS exposure and DBP (*P* value = 0.377).

**Table 3:** Multivariable associations of secondhand smoke concentrations with blood pressure from the National Health and Nutrition Examination Survey, 1999–2016

	Systolic blood pressure		Diastolic blood pressure	
	Beta 95% CI	*P* value	Beta 95% CI	*P* value
Total	0.43 (0.18, 0.67)	0.001	− 0.31 (− 0.51, − 0.12)	0.002
Sex				
Male	0.16 (− 0.14, 0.47)	0.292	− 0.39 (− 0.66, − 0.12)	0.004
Female	0.68 (0.31, 1.05)	< 0.001	− 0.16 (− 0.44, 0.12)	0.261
Race				
Mexican American	0.06 (− 0.47, 0.59)	0.826	− 0.64 (-1.07, − 0.21)	0.003
Other Hispanic	− 0.02 (− 0.84, 0.80)	0.970	− 0.45 (-1.08, 0.19)	0.169
Non-Hispanic white	0.38 (− 0.11, 0.87)	0.125	− 0.23 (− 0.16, 0.14)	0.224
Non-Hispanic black	0.54 (0.14, 0.95)	0.009	− 0.22 (− 0.56, 0.11)	0.195
Other race—including multiracial	0.62 (− 0.20, 1.43)	0.140	− 0.31 (− 0.98, 0.36)	0.359
BMI category				
< 25	0.31 (− 0.02, 0.65)	0.065	− 0.31 (− 0.60, − 0.04)	0.026
25–30	0.74 (0.23, 1.25)	0.004	− 0.09 (− 0.50, 0.32)	0.467
≥ 30	0.23 (− 0.23, 0.69)	0.324	− 0.48 (− 0.84, − 0.12)	0.008
Family PIR				
< 1	0.47 (0.03, 0.91)	0.036	− 0.09 (− 0.45, 0.27)	0.629
≥ 1	0.40 (0.11, 0.69)	0.007	− 0.39 (− 0.62, − 0.16)	0.001
Physical activity				
None	0.42 (0.05, 0.78)	0.027	− 0.38 (− 0.67, − 0.09)	0.009
Moderate	0.63 (0.04, 1.23)	0.037	0.11 (− 0.36, 0.57)	0.654
Vigorous	0.28 (− 0.07, 0.63)	0.118	− 0.44 (− 0.74, − 0.13)	0.005
Diabetes history				
No	0.38 (0.14, 0.61)	0.002	− 0.34 (− 0.53, − 0.15)	0.001
Yes	1.63 (− 0.09, 3.36)	0.064	0.78 (− 0.47, 2.03)	0.220
Alcohol consumption				
No	0.72 (0.06, 1.38)	0.032	− 0.14 (− 0.64, 0.35)	0.575
Yes	0.74 (0.31, 1.18)	0.001	− 0.19 (− 0.52, 0.15)	0.279
CVD history				
No	0.80 (0.43, 1.18)	< 0.001	− 0.11 (− 0.40, 0.17)	0.437
Yes	0.63 (-1.18, 2.45)	0.493	− 0.82 (-2.04, 0.39)	0.182

*CI*, confidence interval; *BMI*, body mass index; *PIR*, poverty-to-income ratio; *CVD*, cardiovascular disease

The result was obtained after adjusting for age, sex, race, PIR, BMI, physical activity, diabetes, alcohol consumption, estimated glomerular filtration rate, CVD, and NHANES cycle

## Discussion

For the first time, Alshaarawy et al. used cotinine levels to quantify SHS exposure and found that SHS exposure was associated with hypertension risk and SBP [37]. Our results are consistent with these results: using a larger sample size, we also found that there was a nonlinear relationship between SHS exposure and SBP through RCS analysis. The stratified analysis results showed that women, non-Hispanic black subjects, those with a PIR greater than 1, individuals with no CVD, and individuals with no diabetes were more likely to be exposed to SHS and have high BP, although there was no interaction between SHS and the above factors.

Although some studies also analyzed the relationship between hypertension and SHS [30, 32], our study focused on the association between SHS and BP and included individuals who were considered healthy. Data from the NHANES showed that the level of serum cotinine in people exposed to SHS was approximately 30 times that in those without SHS exposure. In addition, serum cotinine can be a sensitive indicator of the levels of exposure to SHS. Therefore, in this study, the levels of serum cotinine in nonsmokers represented levels of exposure to SHS instead of the number of cigarettes or hours of exposure per day. Additionally, after excluding those who smoked, 26,578 nonsmokers with SHS exposure were enrolled, without focusing only on one sex or family exposure. Our results provide more objective and rigorous evidence of the relationship between SHS and BP.

Our results found that more women, non-Hispanic black subjects, a PIR greater than 1, those with no CVD, and those with no diabetes were more likely to have SHS exposure and hypertension. There are sex differences in the occurrence of hypertension [43]. Sex differences in BP may also be related to the effect of the hormone environment on long-term BP regulating systems (such as the renin-angiotensin system in adulthood) [44]. Decreases in estrogen can cause an increase in hypertension in females [45]. It has been reported that exposure to SHS can reduce estrogen in mice [46], which may explain women's susceptibility to SHS and hypertension. Our results also found that non-Hispanic black people are susceptible to secondhand smoking and hypertension, and non-Hispanic black people have been shown to be a high-risk group for hypertension in the American population [47]. In addition, previous studies [48] and our results show that non-Hispanic Black subjects have the highest exposure to SHS. This may explain why non-Hispanic black subjects are susceptible to secondhand smoking and hypertension. The high-income group was also susceptible to SHS and hypertension. There is no mechanism-related research to support this hypothesis, and more research is needed. Diabetes and CVD are closely related risk factors for hypertension. However, in our study, a stratified analysis of these two diseases revealed that people without diabetes and CVD were susceptible to SHS and hypertension. However, more research is warranted to explain this result.

We also observed a negative association between SHS and DBP. Several studies investigating the relationship between exposure to environmental pollutants (other than SHS) and BP have reported elevated SBP, with no similar increase in DBP in subjects [49, 50]. However, the exact biological mechanism is unknown. In mice, cigarette smoke exposure can upregulate both SBP and DBP [51–53]. In addition, elevated SBP, but not DBP, was detected in some mice exposed to cigarette smoke [54–56]. Moreover, SBP is more important than DBP for assessing CVD risk [37].

The mechanism by which SHS induces elevated SBP remains unclear, although we still speculated on the possible mechanism based on current studies. Vasodilatation dysfunction, autonomic nervous system imbalance, and vascular aging stimulated by particulates, nicotine, and other components are considered potential mechanisms. First, numerous studies have shown that SHS is involved in the regulation of endothelial function [57] and leads to systemic vascular oxidative stress with increasing reactive oxygen species (ROS) and decreasing nitric oxide (NO) [11]. However, it is worth noting that elevated ROS and reduced NO could influence vasodilatation function and further increase BP and pulse pressure [58, 59]. Second, nicotine from SHS could release catecholamine and influence sympathetic-vagal balance. Although this stimulation was transient [60], repeated exposure to SHS may result in autonomic nervous system disorder and subsequent vascular remodeling [61, 62], which could increase BP. Third, SHS could also accelerate vascular aging [63, 64], which could increase ROS production, decrease biological utilization of NO and further affect BP.

Some limitations exist in our research. First, although serum cotinine can more precisely reflect the extent of exposure to SHS, other components should also be considered, such as fine particulate matter (PM) and acrolein, which are involved in endothelial damage and further affect BP. In addition, the effect of third-hand smoke (THS), which is composed of nicotine along with some novel components and is produced by aging and the accumulation of SHS [65, 66], on BP should also have been considered in our study. However, it was difficult to estimate the influence of THS on BP due to the lack of accurate THS data in NHANES. In addition, genetic susceptibility, such as family history [67, 68], and environmental factors, such as place of residence, can be confounding factors that may affect the association results, but this data was not available in NHANES. However, we cannot ignore the influence of these factors.

## Conclusion

Our study found that a high level of SHS was positively related to increased SBP in adults. The underlying mechanism of SHS-induced hypertension may involve vasodilatation dysfunction, vascular remodeling regulated by the autonomic nervous system, and vascular aging.

## Supplementary Information

**Additional file 1: Figure S1.** The distribution of serum cotinine levels.

**Additional file 2: Table S1.**

## Data Availability

All data generated or analyzed during this study are included in this published article and its supplementary information files.

## Abbreviations

BP: Blood pressure
SHS: Secondhand smoke;
NHANES: National Health and Nutrition Examination Survey
PIR: Poverty-to-income ratio
BMI: Body mass index
SBP: Systolic blood pressure
DBP: Diastolic blood pressure
CVD: Cardiovascular disease
MEC: Mobile examination center
ID HPL-APCI MS/MS: Isotope dilution high-performance liquid chromatography/atmospheric pressure chemical ionization tandem mass spectrometry
RCS: Restricted cubic spline
ROS: Reactive oxygen species
NO: Nitric oxide
PM: Particulate matter
THS: Third-hand smoke
