# Supplementary figures and images for: Associations of exposure to secondhand smoke with hypertension risk and blood pressure values in adults
Source: Environ Health Prev Med. 2021 Sep 6;26:86. doi: 10.1186/s12199-021-01009-0 (PMC8422707; doi:10.1186/s12199-021-01009-0)

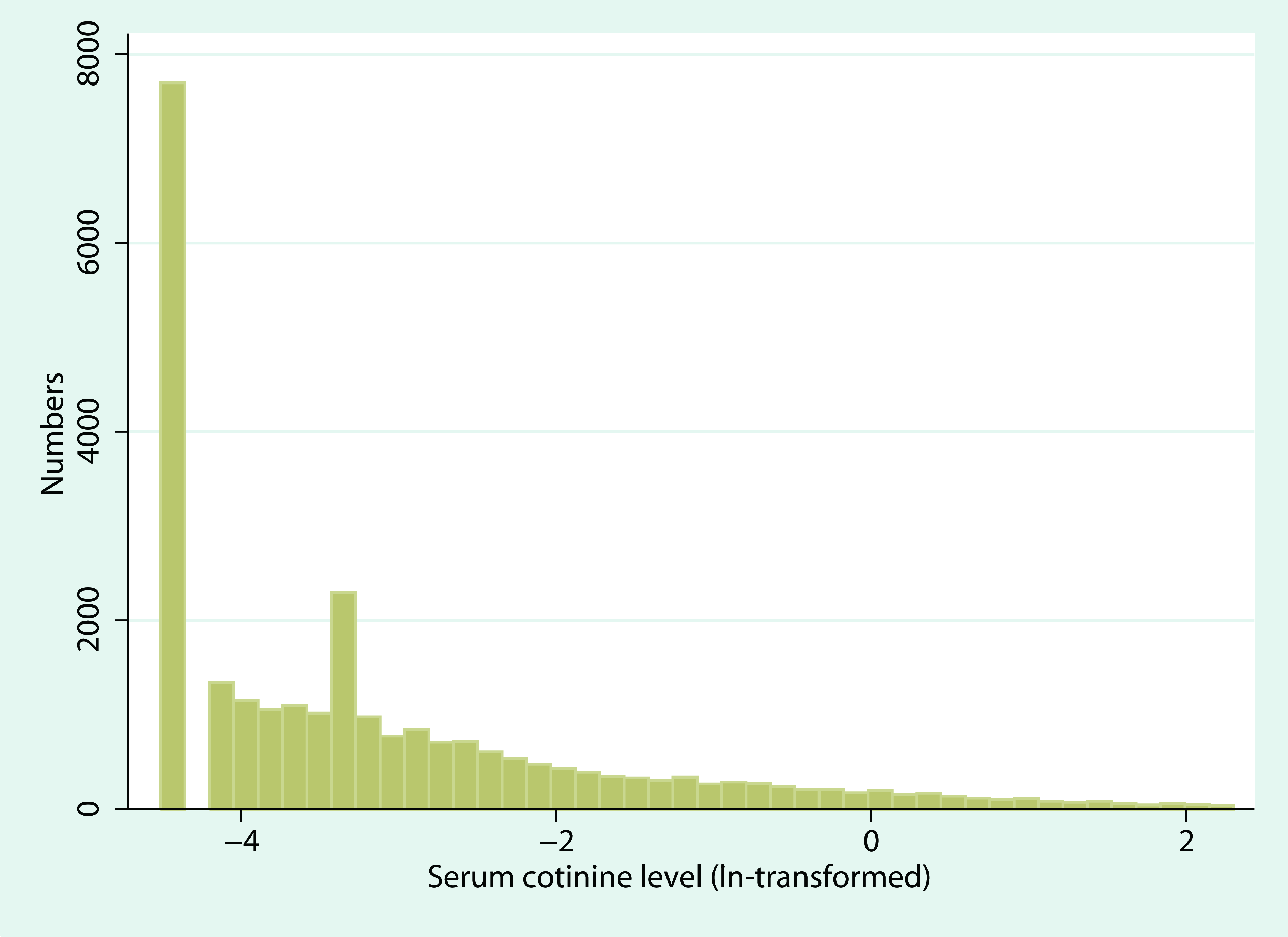

Supplement: Supplementary file 1 — Additional file 1: Figure S1. The distribution of serum cotinine levels. [file 12199_2021_1009_MOESM1_ESM.tif]
