# Supplementary material for: Associations of exposure to secondhand smoke with hypertension risk and blood pressure values in adults
Source: Environ Health Prev Med. 2021 Sep 6;26:86. doi: 10.1186/s12199-021-01009-0 (PMC8422707; doi:10.1186/s12199-021-01009-0)
Supplement: Supplementary file 2 — Additional file 2: Table S1. [file 12199_2021_1009_MOESM2_ESM.docx]

**Table S1.** ORs for the associations between secondhand smoke concentrations and hypertension risk stratified by covariates in the National Health and Nutrition Examination Survey, 1999–2016

|  | OR (95% CI) | P value |
| --- | --- | --- |
| SHS |  |  |
| Tertiles 1 | Reference |  |
| Tertiles 2 | 1.05 (0.96, 1.13) | 0.295 |
| Tertiles 3 | 1.13 (1.04, 1.24) | 0.007 |
| Sex |  |  |
| Male | Reference |  |
| Female | 1.13 (1.05, 1.22) | 0.001 |
| Race |  |  |
| Mexican American | Reference |  |
| Other Hispanic | 1.16 (1.02, 1.33) | 0.027 |
| Non-Hispanic white | 1.05 (0.95, 1.16) | 0.349 |
| Non-Hispanic black | 2.00 (1.80, 2.23) | <0.001 |
| Other race - including multiracial | 1.36 (1.18, 1.57) | <0.001 |
| BMI category |  |  |
| <25 | Reference |  |
| 25–30 | 1.91 (1.74, 2.09) | <0.001 |
| >=30 | 3.53 (3.23, 3.87) | <0.001 |
| Family PIR |  |  |
| <1 | Reference |  |
| >=1 | 0.93 (0.85, 1.02) | 0.148 |
| Physical activity |  |  |
| None | Reference |  |
| Moderate | 1.02 (0.94, 1.10) | 0.703 |
| Vigorous | 0.96 (0.88, 1.06) | 0.433 |
| Diabetes history |  |  |
| No | Reference |  |
| Yes | 2.58 (2.33, 2.86) | <0.001 |
| Alcohol consumption |  |  |
| No | Reference |  |
| Yes | 1.01 (0.94, 1.09) | 0.836 |
| CVD history |  |  |
| No | Reference |  |
| Yes | 2.17 (1.92, 2.46) | <0.001 |
| NHANES cycle |  |  |
| 1999-2000 | Reference |  |
| 2001-2002 | 0.87 (0.74, 1.03) | 0.099 |
| 2003-2004 | 0.92 (0.78, 1.09) | 0.354 |
| 2005-2006 | 0.93 (0.79, 1.10) | 0.379 |
| 2007-2008 | 0.93 (0.80, 1.09) | 0.377 |
| 2009-2010 | 1.03 (0.89, 1.21) | 0.674 |
| 2011-2012 | 1.01 (0.86, 1.18) | 0.949 |
| 2013-2014 | 1.12 (0.96, 1.32) | 0.153 |
| 2015-2016 | 1.01 (0.86, 1.19) | 0.874 |

OR, odd ratio. CI, confidence interval. SHS, secondhand smoke. BMI, body mass index. PIR, poverty-to-income ratio. CVD, cardiovascular disease. NHANES, National Health and Nutrition Examination Survey.

The result was analysed after adjusting for age, sex, race, PIR, BMI, physical activity, diabetes, alcohol consumption, estimated glomerular filtration rate, CVD, and NHANES cycle.
